# Multisystem Inflammatory Syndrome in a Young Adult (MIC-A) Following SARS-CoV-2 Infection

**DOI:** 10.3390/medicina58111515

**Published:** 2022-10-24

**Authors:** Ausrine Bajoriunaite, Jurgita Zaveckiene, Akvile Usaite, Olivija Dobiliene, Tomas Tamosuitis

**Affiliations:** 1 Department of Intensive Care, Medical Academy, Lithuanian University of Health Sciences, LT-44307 Kaunas, Lithuania; 2 Department of Radiology, Medical Academy, Lithuanian University of Health Sciences, LT-44307 Kaunas, Lithuania; 3 Hospital of Lithuanian University of Health Sciences, LT-50161 Kaunas, Lithuania; 4 Department of Cardiology, Medical Academy, Lithuanian University of Health Sciences, LT-44307 Kaunas, Lithuania

**Keywords:** multisystem inflammatory syndrome, SARS-CoV-2, intravenous immunoglobulin, anti-IL-6 antibodies

## Abstract

Multisystem Inflammatory Syndrome (MIS) is a rare but increasingly recognized complication of SARS-CoV-2 infection, usually presenting 2 to 6 weeks after the onset of COVID-19 infection symptoms and affecting mainly children. However, there have been reported several cases of a similar multisystem inflammatory syndrome in adults (MIS-A). We describe the case of a previously healthy 28-year-old male who presented with a clinical profile with multiorgan involvement within four weeks after confirmed SARS-CoV-2 infection, suggestive for multisystem inflammatory syndrome (MIS-A). The clinical presentation included persistent high grade of fever, gastrointestinal and mucocutaneous lesions, lymphadenopathy, elevated cardiac and inflammatory biomarkers, cytopenia and shock. This case report illustrates the wide range of presentations, diagnosis, and treatment modalities of multisystem inflammatory syndrome. The pathophysiology and the mechanisms by which SARS-CoV-2 triggers an abnormal immune response leading to MIS remain poorly understood. Better characterization of MIS-A and early recognition of MIS is important because it is associated with high mortality if left untreated.

## 1. Introduction

Multisystem inflammatory syndrome (MIS) is a rare but severe, potentially life-threatening condition, presenting from 2 to 6 weeks after the onset of COVID-19 infection symptoms, and affecting mainly children [1,2]. Since June 2020, several case reports have been published about multisystem inflammatory syndrome in adults (MIS-A) with a broad spectrum of clinical symptoms and treatment options [3,4]. The pathophysiology and the mechanisms of MIS-A are not known; however, it is suspected to be caused by dysregulated immune response to severe acute respiratory syndrome coronavirus 2 (SARS-CoV-2) infection, and usually manifests with fever, systemic inflammation and multiple organ system involvement [5]. It is difficult to diagnose MIS because of the occurrence of other types of COVID-19-related hyperinflammation, which makes MIS hard to distinguish from biphasic acute COVID-19 and post-acute sequelae of SARS-CoV-2 infection [6,7,8]. Moreover, MIS in children (MIS-C) after COVID-19 shares many similarities with other inflammatory diseases, such as Kawasaki disease and toxic shock syndrome, associated with a cytokine storm suggestive of a macrophage activation syndrome, therefore it can create difficulties to differentiate between them [9,10]. It is of great importance to properly diagnose and start treating MIS-A, because uncontrolled MIS has high mortality.

## 2. Case Presentation 

**Figure 1:**
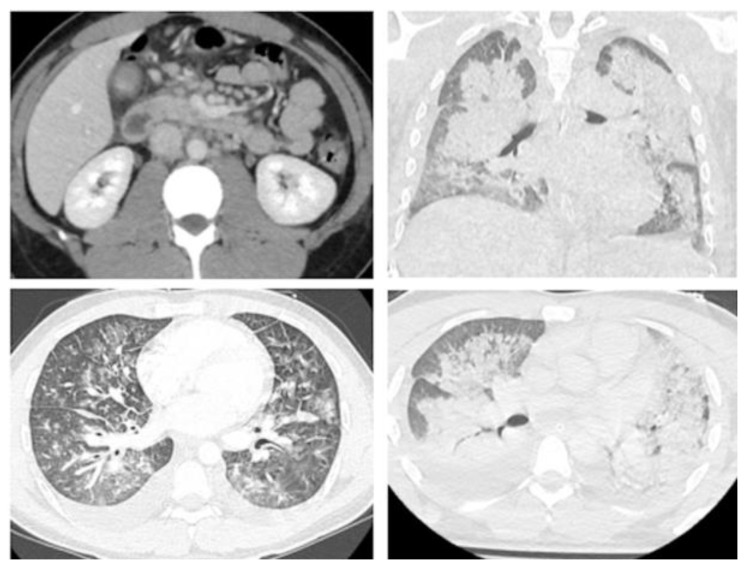
Computer tomography images (**Left**—day 1, **Right**—day 5): intraabdominal lymphadenopathy; pleural effusion, septal thickening, ground glass opacification and progressive parenchymal consolidation.

**Figure 2:**
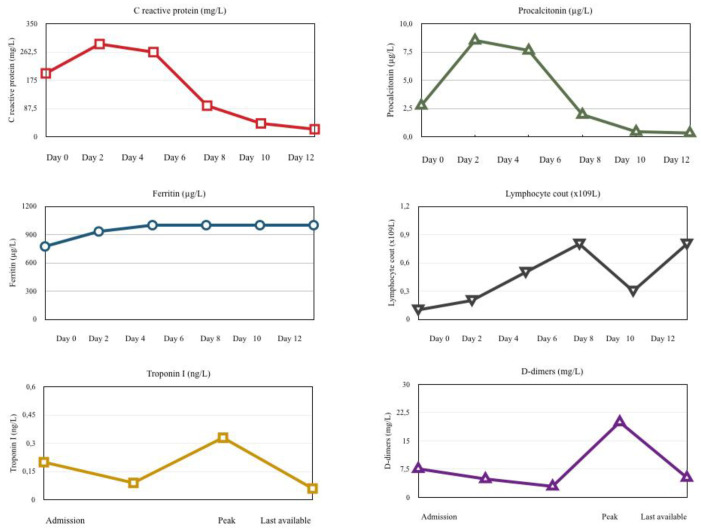
Inflammatory markers in peripheral blood of patient during the treatment.

The patient was a 28-year-old previously healthy man presented to the emergency department in the Hospital of Lithuanian University of Health Sciences with high-grade fever (39–40 °C), persistent for five days, acute abdominal pain, vomiting and diarrhea, which, after 3 days, were followed by: maculopapular rash on patient’s trunk and extremities, neck swelling, lymphadenopathy and bilateral conjunctival injections. Six weeks prior to these clinical manifestations, the patient was infected with a mild SARS-CoV-2 infection and completely recovered within 1 week. After a few hours in the emergency department, he was admitted to an intensive care unit for treatment of hemodynamic instability. New-onset heart failure and vasoplegia that progressed into shock with multiorgan dysfunction were diagnosed. The patient received supportive treatment with dynamic hemodynamic-driven preload resuscitation, complex vasopressor and inotrope support. He developed a severe hypoxic respiratory failure due to pulmonary edema which required mechanical ventilation (Figure 1). Laboratory testing revealed high levels of inflammatory markers, including C-reactive protein, procalcitonin, ferritin, D-dimer, troponin-I, B-type natriuretic peptide and leukocytosis, with a predominance of neutrophils, lymphopenia and thrombocytopenia (Table 1). Because of suspected gastrointestinal tract infection, the patient received broad spectrum empirical antibiotic therapy (vancomycin, meropenem). However, all tests for bacteria (urine, blood culture) were negative. Despite this antibiotic therapy, the patient’s clinical state gradually worsened. After this, acute renal failure was additionally detected. In the absence of a more likely alternative diagnosis (infections, oncologic and autoimmune/inflammatory conditions) he was diagnosed and treated for MIC-A. Inflammatory markers in peripheral blood during treatment and medications used are presented in Figure 2. Treatment of the acute kidney injury and cytokine removal using continuous veno-venous hemofiltration with sorbent adsorption was started on hospital Day 2. On the same day, intravenous immunoglobulin (IVIG) infusion was initiated and completed. Patient showed resistance to IVIG and received a high dose of glucocorticosteroid (GCS), which was completed on Day 6. A continuous fever, no improvement in clinical status and persistent severe condition with heart, pulmonal and renal failure, followed by high inflammatory markers, demonstrated inadequate treatment. On Day 7, despite prophylaxis with antiplatelet and anticoagulant therapies, pulmonary embolism (PE) was diagnosed and low molecular weight heparin was added to treat it. Moreover, it was decided to even add cytokine-target therapy, monoclonal anti-IL-6 receptor antibody. The patient’s clinical status and laboratory tests normalized gradually. By Day 8 he was weaned off vasopressors, and on Day 18 discharged home on continuous treatment with direct acting oral anticoagulant and low dose of GCS.

Treatment at Day 2: intravenous immunoglobulin (2 g/kg, total dose 160 g) IV; broad spectrum antibiotics (vancomycin, meropenem); inotropic and vasoactive treatment (norepinephrine, vasopressin, dobutamine); fluid resuscitation; respiratory support; continuous veno-venous hemofiltration (with sorbent adsorption); antiplatelet and anticoagulant therapies (prophylaxis). Treatment at Day 4: GCS was added, methylprednisolone (6 mg/kg/day, total dose 500 mg for 3 days) IV. Treatment at Day 7: anti-IL-6 monoclonal antibody was added, tocilizumab (8 mg/kg, total dose 664 mg) IV; broad-spectrum antibiotics (colistin, cefoperazone/sulbactam); anticoagulants for PE (low molecular weight heparin—nadroparine 5700 anti Xa IU x2 SQ). 

IV—intravenous infusion; SQ—subcutaneous injection; IVIG—intravenous immunoglobulin; GCS—glucocorticosteroid; anti-IL-6 monoclonal antibody, PE—pulmonary embolism.

## 3. Discussion

Multisystem inflammatory syndrome (MIS) is a rare complication of SARS-CoV-2 infection, usually presenting from 2 to 6 weeks after the onset of the COVID-19 infection symptoms and affecting mainly children. It is now known that adult patients with current or previous SARS-CoV-2 infection can develop a hyperinflammatory syndrome [12,13]. The diagnostic criteria are fever, elevated markers of inflammation, and multisystem involvement without an alternative diagnosis [11]. In this case, the patient had high-grade fever, skin rash, conjunctivitis, and lymphadenopathy. He also had an acute abdominal pain for which was consulted by surgeons, but no pathology was found. However, lymphadenopathy and a small amount of liquid were revealed by computed tomography of the abdomen. At the beginning of the disease, the patient neither had respiratory symptoms nor respiratory failure (nor typical viral changes in CT of the lungs, which is common for a severe case of COVID-19 disease). Extrapulmonary symptoms were severe and progressed quickly, affecting other organs under a short period of time. Shortly afterwards, the failure of the cardiovascular system, lungs and kidneys was noted. The COVID-19 antigen test from the nasopharyngeal swab specimen, which was performed several times, was negative, in comparison to the previous test 6 weeks ago, which was positive (with the SARS-CoV-2 infection). This period of time between SARS-CoV-2 infection and occurred hyperinflammation allowed us to suspect MIS-A.

MIS of COVID-19 shares many similarities with other inflammatory diseases, such as Kawasaki disease and toxic shock syndrome, associated with a cytokine storm suggestive of a macrophage activation syndrome, therefore it can create great difficulty to differentiate between them [14,15]. The key feature that would help to distinguish between these diseases is a positive antibody test after COVID-19 infection. It is of a great importance to properly diagnose each one of these conditions, because the treatment differs. If MIS and other overlapping hyperinflammatory syndromes were undiagnosed or the treatment was delayed, it could cause life-threatening complications and is associated with high mortality.

The pathophysiology of MIS-A is complex and involves endothelial damage, thromboinflammation, immune dysregulation (dysregulating the innate and adaptive host immune responses) as well as the renin-angiotensin-aldosterone system, and/or viral persistence in extra-pulmonary tissues [16]. The data from children MIS-C studies suggests that patients with severe MIS have persistent immunoglobulin G-antibodies with enhanced ability to activate monocytes, persistent low count of T-lymphocytes and activation of CD8+ T cells [17,18].

Patients with SARS-CoV-2-associated MIS respond to immune modulation with IVIG and GCS and show increased inflammatory markers, thus the disease is likely immunologically mediated. According to the guidelines from the American College of Rheumatology [2], the treatment of MIS goals are to stabilize patients with life-threatening manifestations, such as shock, and prevent long-term consequences, such as coronary artery dilation or coronary artery aneurysms, or myocardial fibrosis/scarring. Since our discussed patient’s condition was critical, supportive care and early initiation of immunomodulatory treatment with a high dose of IVIG (2 mg/kg) was needed. Immunomodulatory medications used in treating MIS-C patients were both IVIG and GCS. It is difficult to compare the efficacy of IVIG vs. GCS, or whether these medications should be provided individually or as a dual therapy in MIS-C, in consequence of the lack of data available. Due to the lack of response to treatment with IVIG, our patient persisted with shock and thus required multiple inotropes and/or vasopressors. The patient was also being treated with a high dose of GCS for 72 h. The patient’s condition, clinical organ failure symptoms, and inflammatory markers did not improve. In addition to that, abnormalities in the coagulation cascade, including prominent elevations in D-dimer, occurred, and pulmonary embolism was detected with CT of the chest. Deep vein thrombosis or pulmonary embolism appears to be a significant complication of MIS associated with SARS-CoV-2 infection in children. Patients with MIS-C and TE appear to have significant morbidity and/or mortality [19]. The high rates of thrombotic complications can be possibly contributed by the untempered inflammation, along with hypoxia and direct viral-mediated effects. Although the underlying mechanisms of the coagulopathy in COVID-19 and MIC are still unknown, anticoagulation and antiplatelet therapy are generally recommended. There are reports that point out the high rate of TE complications in patients even with respective hypercoagulation prophylaxis or treatment. Analysing the data of anticoagulated severe COVID-19 patients in intensive care unit, a 69% incidence rate of venous TE complications was detected [20]. Moreover, the article also points out the high rate of TE events in COVID-19 patients treated with therapeutic anticoagulation, with 56% of venous TE and 6 PE. It is of a great importance to further research the mechanisms and treatment of coagulopathy in COVID-19 and MIC, in order to be able to choose appropriate anticoagulants, their dose and duration of the treatment. IL-1, IL-6, and TNF inhibitors have been used as a second-line therapy in patients with MIC-C who are refractory to initial therapy with IVIG and low- to moderate-dose glucocorticoids. A large study in the USA on MIS-C shows that 80–100 percent of patients have increased serum concentrations of IL-6 [21], therefore mention the successful use of tocilizumab in controlling inflammation [4]. Tocilizumab suppresses fever and acute phase reactants, such as C-reactive protein and ferritin, and in this case, since we could not measure the changes of serum concentrations of IL-6, we were evaluating the dynamic of the inflammatory markers and clinical manifestations instead. In theory, IL-1 is found upstream of IL-6 in inflammatory cascades, thus effective inhibition of IL-1 in COVID-19 patients with hyperinflammation may result in more robust clinical benefits. There is not enough information about the profile of the optimal candidates for treatment with interleukin inhibitors, however, according to another study, IL-6 inhibition was associated with a significant reduction of mortality in patients admitted to hospital with COVID-19, respiratory insufficiency, and hyperinflammation in a subgroup of patients with markedly high C-reactive protein and low lactate dehydrogenase concentrations [22]. According to the international guidelines, anti-IL-6R drugs are also recommended as one of the options available to severe or critically ill COVID-19 patients with hyperinflammatory response [23]. In our case, treatment with tocilizumab showed a good response, the patient’s clinical condition improved considerably, and cardiac function was partially restored. In order to avoid the relapse and long-term complications of MIS after ceasing, low-dose GCS was prescribed for 4 weeks together with antithrombotic therapy using the oral anticoagulant rivaroxaban.

Talking about COVID-19 vaccination, current knowledge shows that COVID-19 vaccination is the best protection from MIS [24]. Although there is not enough information about the safety of COVID-19 vaccines in patients who have had MIS-C or MIS-A, experts discuss that the benefits of COVID-19 vaccination for people with a history of MIS-C/A outweighs a theoretical risk of an MIS-like illness following COVID-19 vaccination for those who meet the following two criteria: full clinical recovery has been achieved (including return to baseline cardiac function) and at least 90 days after the diagnosis of MIS-C/A [25]. 

## 4. Conclusions

These findings suggest that MIS-A occurs in the post-acute COVID-19 period with a heterogeneous clinical presentation, likely owing to a dysregulated immune response. Prompt immune-modulatory treatment is needed in order to stabilize the disease and to avoid severe and life-threatening complications. Uncontrolled MIS has a high mortality, and it is of great importance to properly and early diagnose and start the treatment. The importance of vaccination among all eligible persons is based on the growing evidence that COVID-19 vaccination is associated with a high level of protection against MIS in children and adults.

## Figures and Tables

**Table 1:** Clinical and biological characteristics of the patient (on the day of admission to the hospital (emergency unit)).

Characteristics	Values (Reference Values)
I. MIC-A clinical criteria *	
Documented persistent fever (≥38.0 °C) and at least 3 of the following clinical criteria **:	Yes
A. Primary clinical criteria	
1. Severe cardiac illness	Yes
2. Rash and non-purulent conjunctivitis	Yes
B. Secondary clinical criteria	
1. New-onset neurologic signs and symptoms	No
2. Shock or hypotension not attributable to medical therapy	Yes
3. Abdominal pain, vomiting, or diarrhea	Yes
4. Thrombocytopenia	Yes
II. MIC-A laboratory evidence	
The presence of laboratory evidence of inflammation and SARS-CoV-2 infection ***	Yes
Chest radiography/computer tomography	Bilateral interstitial densities and pleural effusions.
Ultrasound findings on heart	Tachycardia 120 beats/min, mild systolic dysfunction, a pericardial effusion.
Laboratory findings	
Nasopharyngeal SARS-CoV-2 PCR	Negative
Serum SARS-CoV-2 IgG levels (BAU/mL)	75.4 (<=21)
Leukocyte count (×10^9^ L)	19.4 (3.6–10.2)
Lymphocyte count (×10^9^ L)	0.1 (1.1–3.2)
Neutrophil count (×10^9^ L)	19 (1.7–7.6)
Thrombocyte count (×10^9^ L)	140 (152–348)
C reactive protein (mg/L)	196 (0–5)
Procalcitonin (µg/L)	2.76 (0–0.1)
D-dimers (mg/L)	7.6 (0–0.5)
Ferritin (µg/L)	774 (25–380)
Troponin I (ng/L)	0.2 (0–0.04)
B-type natriuretic peptide (ng/L)	248 (0–26.5)
LDH (U/L)	326 (100–250)

* The Centre for Disease Control and Prevention (CDC) recommendations for MIC-A diagnosis [11]. ** Subjective or documented fever (≥38.0 °C) for ≥24 h prior to hospitalization or within the first 3 days of hospitalization and at least 3 of the following clinical criteria occurring prior to hospitalization or within the first 3 days of hospitalization. At least one must be a primary clinical criterion. Thrombocytopenia is defined as a platelet count of less than 150 × 10^9^/L. *** Elevated levels of at least 2 of the following: C-reactive protein, ferritin, IL-6, erythrocyte sedimentation rate, procalcitonin. PCR—polymerase chain reaction; SARS-CoV-2—severe acute respiratory syndrome coronavirus; IgG—immunoglobulin G; LDH – lactate dehydrogenase; MIC-A—multisystem inflammatory syndrome in adults.

## Data Availability

Not applicable.
